# Hereditary Neuroses in Children.—VI

**Published:** 1897-06-26

**Authors:** 


### Modern Sociology.

### HEREDITARY NEUROSES IN CHILDREN.—VI.

Having in preceding papers glanced at the various modes in which children may be affected by neurotic inheritance we have now to inquire, Whence does this neurotic inheritance originate, and in what directions is it transmissible? To trace the genesis of a neurosis is no easy matter. Unfavourable environment may in some cases act as a factor of mental degeneracy, as well as of physical. A sturdy race of mountaineers, driven by stress of circumstances to reside in deep valleys, uncheered by the direct rays of the sun, gradually lose vigour, and, as may be seen in some parts of Switzerland, become stunted in body and enfeebled in mind. Intermarrying, their progeny (also exposed to like unfavourable surroundings) become cretinous, and the morbid type so evolved is intensified from generation to generation. Impressions derived from dull and squalid surroundings, constantly registered on the brain—as in the case of dwellers in insanitary slums—give rise not only to an effect of dulness in the individual, but abiding effects of such impressions may pass to another generation. There comes into play, moreover, in the majority of such cases, another factor of degeneration in the form of drink, taken to at first, perhaps, as a delusive antidote to dulness. Alcohol specially attacks the higher nerve centres, weakening the powers of self-restraint, and so a vicious circle is established. In the preceding paper a pedigree was quoted from Morel, in which varied forms of neuroses appeared in the grandchildren of a drunkard of originally healthy stock. But it is not only alcoholic toxæmia, but other infective processes, associated with bacterial toxines, that may be followed by psychoses and neuroses of a transmissible character. Thus Putnam* states that out of 5,000 patients suffering from affections of the nervous system he found that 500 had had, within a year of the onset of the illness, some infectious disease, such as influenza, syphilis, diphtheria, typhoid, scarlatina, or malaria. Gout and rheumatism also appear to have a special affinity with certain heritable nervous affections, such as chorea and neuralgia. But in addition to infections through the blood, it is probable, as Maudsley† has remarked, that habitual "thoughts, feelings, and actions leave behind them certain residua which become organised in the nervous centres, and thenceforth modify the manner of their development or constitute their acquired nature."

We see, therefore, that a previously healthy stock, exposed to unfavourable influences either from without or within, may take on an unhealthy mode of nerve action; in other words, develop a neurosis. Once developed, this acquired habit of disorderly action becomes a second nature, liable to transmission to descendants as a *damnosa hæreditas*. Not necessarily is the form of neurosis the same in the child as in the parent. The drunkard does not always bequeath drink-craving to his son, but too often endows him with a nervous instability which incapacitates him from holding his own in the battle of life, and he falls a victim to the "slings and arrows of outrageous fortune." If not actually insane he may be a recognised "eccentric." In youth, indeed, he may even pose as a prodigy for a time; but in the long run he turns out a social failure. In his progeny, if he has any, neuroses show themselves in various forms; one child may be epileptic, another mentally defective, whilst a third may display the artistic temperament in a marked degree, and a fourth be a moral imbecile. This, of course, is more or less of a fancy picture, but similar cases are sometimes met with in real life. The records of idiot asylums furnish a number of "awful examples" in the family history of the inmates, and it may be instructive to quote a few actual* cases throwing light upon the etiology of mental defect in children. Case 1: "Both parents intemperate; paternal aunt and two maternal aunts died of phthisis; brother very excitable." Case 2: "Father's uncle insane; father insane (after sunstroke); three brothers and one sister of father died of phthisis; two brothers also imbecile!" Case 3: "Mother phthisical, father intemperate; parents first cousins; brother idiot." Such cases serve to show (*inter alia*) how neuroses are not only transmissible but transmutable in a neurotic family, and how imprudent marriages may intensify a predisposition to nervous disease.

Fortunately, there is another and less discouraging aspect of the subject of environment and heredity to be considered. If gloomy and insanitary surroundings tend to produce neurosis, the converse is also true that, given a tendency to neurosis, bright and healthy surroundings will keep it in check. So, also, if Nature is allowed a fair chance, and not handicapped by the piling up of morbid heredity, the law of reversion to the normal type will assert itself, and an ancestral neurotic taint will gradually be obliterated. It is rarely, however, that those who know (or ought to know) that they inherit a predisposition to nervous weakness give to this matter as grave consideration as is due, and consequently the "vis medicatrix naturæ" has but limited scope. Moreover, there seems to be a sympathetic affinity between persons of neurotic temperament which leads to intermarriage and intensification of the strain. Although once in a way the product may be a great preacher, a great painter, a great poet, or a great musician (for this temperament tends to give prominence to the æsthetic faculties), as a rule the risk of disaster is great, and at best the progeny of neurotic parents will be short of intellectual "staying power," even if they escape the grosser ills which have formed the subject of these papers.

* Amer. Jour. Med. Science. 1895. p. 254.
† Physiology and Pathology of Mind, p. 200 (1st edit.).

* From Case-books, Royal Albert Asylum.

